# Quantum dynamics studies of isotope effects in the Mg^+^(3p) + HD → MgH^+^/MgD^+^ + D/H insertion reaction

**DOI:** 10.1038/s41598-020-60033-2

**Published:** 2020-02-25

**Authors:** Ye Mao, Jiuchuang Yuan, Zijiang Yang, Maodu Chen

**Affiliations:** Key Laboratory of Materials Modification by Laser, Electron, and Ion Beams (Ministry of Education), School of Physics, Dalian University of Technology, Dalian, 116024 PR China

**Keywords:** Reaction kinetics and dynamics, Molecular dynamics

## Abstract

The time-dependent wave packet quantum dynamics studies for the Mg^+^(3p) + HD → MgH^+^/MgD^+^ + D/H diabatic reaction are carried out for the first time on recently developed diabatic YHWCH potential energy surfaces [Phys. Chem. Chem. Phys., 2018, 20, 6638–6647]. The results of reaction probabilities and total integral cross sections show a dramatic preference to the formation of MgD^+^ over MgH^+^ owing to the insertion reaction mechanism in the title reaction. The MgD^+^/MgH^+^ branching ratio witnesses a monotonic decrease from 10.58 to 3.88 at collision energy range of 0.01 to 0.20  eV, and at the collision energy of 0.114  eV, it is close to the experimental value of 5. The rovibrational state-resolved ICSs of the two channels show the products MgD^+^ have higher vibrational excitation and hotter rotational state distributions. The opacity function P(*J*) suggests that the MgH^+^ + D channel and MgD^+^ + H channel are dominated by high-*b* and low-*b* collisions, respectively. Both forward and backward scattering peaks are found in the differential cross section curves, whereas the angle distributions of products are not strictly forward-backward symmetric because of the short lifetime of the complex in the reaction.

## Introduction

Isotope effects play a key role in numerous chemical physics studies, which can shed more light on the study of reaction dynamics. Among the isotopic substitution reactions, the simplest type of A + HD has been the focus of sophisticated investigation by comparing different isotope branches^1–10^. In particular, there are keen interests in the reactions of alkaline earth metal ions (X^+^) with HD molecules due to the strong preference of a certain isotope branching^11–13^. Moreover, the interactions of alkaline earth metal ions with hydrogen molecule and its isotopic variants have received substantial investigations both experimentally and theoretically because of the importance in the field of cold and ultracold chemistry. On the experimental side, the reactions of laser-cooled alkaline earth metal ions, such as Be^+^, Mg^+^ and Ca^+^, with hydrogen and its isotopic counterparts were performed in ion trap apparatus, and the cooled ions can be used as coolant to sympathetically cool the products of molecular ions^14–18^. In theory, these reactions are also favorite objects to study cold and ultracold reaction dynamics.

The collisions of Mg^+^ ion with hydrogen molecule and its isotopic variants have received great attention experimentally in the past. Compared with the ground state Mg^+^ ion, the reactions with electronically excited Mg^+^ ion are of particular interest and complex because of the diabatic processes in the reactions. In 2000, Molhave *et al*.^19^ produced the molecular ions MgH^+^(MgD^+^) in a linear Paul trap by the photochemical reactions of Mg^+^(3p^2^P_3/2_) + H_2_ (D_2_) and the molecular ions were cooled sympathetically by Coulomb interaction with laser-cooled Mg^+^ ions. These cold molecular ions are very valuable for many chemical physics contexts. In 2008, Staanum *et al.*^13^ studied the single ion reactions at thermal energies of laser-cooled Mg^+^(3p^2^P_3/2_) with hydrogen molecule and its isotopic variants. The experimental method used in their work could provide a useful reference for other reaction involving rare reaction partners. Moreover, particularly intriguing for their experiment is that the strong isotope effects were observed in the reactions between Mg^+^(3p^2^P_3/2_) and HD molecules. A remarkable preference of the production of MgD^+^ rather than MgH^+^ was detected and the value of branching ratio between MgD^+^ and MgH^+^ was decided as larger than 5. This preferential production of MgD^+^ was also observed in the reaction of ground state Mg^+^ ions with HD molecules in a series of guided ion beam studies^11^.

Advances in the experiments have aroused the interests in the theoretical studies of MgH_2_^+^ system. Because an accurate potential energy surface (PES) is a critical ingredient to study the reactive collision mechanism, several theoretical works have centered around the construction of PES on this system^20–23^. In 2013, Satta *et al*.^22^ constructed the lowest two adiabatic potential energy surfaces which can describe the interaction of Mg^+^(^2^S) and Mg^+^(^2^P) ions with H_2_ molecule. The PESs were based on *ab initio* energy points by using the complete-active-space self-consistent field method with the aug-cc-pvtz basis set. In addition, they provided a qualitative explanation for the formation of MgD^+^ and MgH^+^ ions in the cold ion trap experimentally. Regarding the preference of the production of MgD^+^, they suggested that the isotope effect comes from the mass effects on the kinematics of nonadiabatic process. However, the reaction of the Mg^+^(3p) + HD involves two different adiabatic states, namely, the ground state 1^2^A′ and the first excited state 2^2^A′. Hence, these adiabatic PESs cannot be used for dynamics study on this diabatic reaction because of the lack of treatment for the coupling of the two states. Until recently, our group presented a set of highly accurate global diabatic PESs (YHWCH PESs) of this system^23^. Regarding YWHCH diabatic PESs, *ab initio* energy points were calculated by using the multi-reference configuration interaction method and the neural network method was used to fit the PESs. The method of obtaining diabatic energy matrixes is to transform adiabatic *ab initio* data by using dipole moment operator which could reflect the transition characteristics between the coupled states. And the presence of the spin-orbit coupling is ignored in the PESs. Based on the new PESs, the time-dependent wave packet (TDWP) calculations for the Mg^+^(3p^2^P) + H_2_(X^1^${\sum }_{g}^{+}$) → MgH^+^(X^1^Σ^+^) + H(^2^S) reaction were implemented to study the diabatic reaction dynamics.

As mentioned above, although many experimental studies have been devoted to the isotope effects in the reaction of the Mg^+^(3p) + H_2_, the dynamics calculations of the Mg^+^(3p) + HD → MgH^+^/MgD^+^ + D/H reaction have not been carried out due to the lack of available diabatic PESs. To obtain more detailed dynamics information for this reaction system, especially to study the different dynamical behaviors between the MgH^+^ + D and MgD^+^ + H channels under strong isotope effects and reveal the reaction mechanism, the TDWP calculations are carried out on the YHWCH diabatic PESs in the present work.

## Results

**Figure 1:**
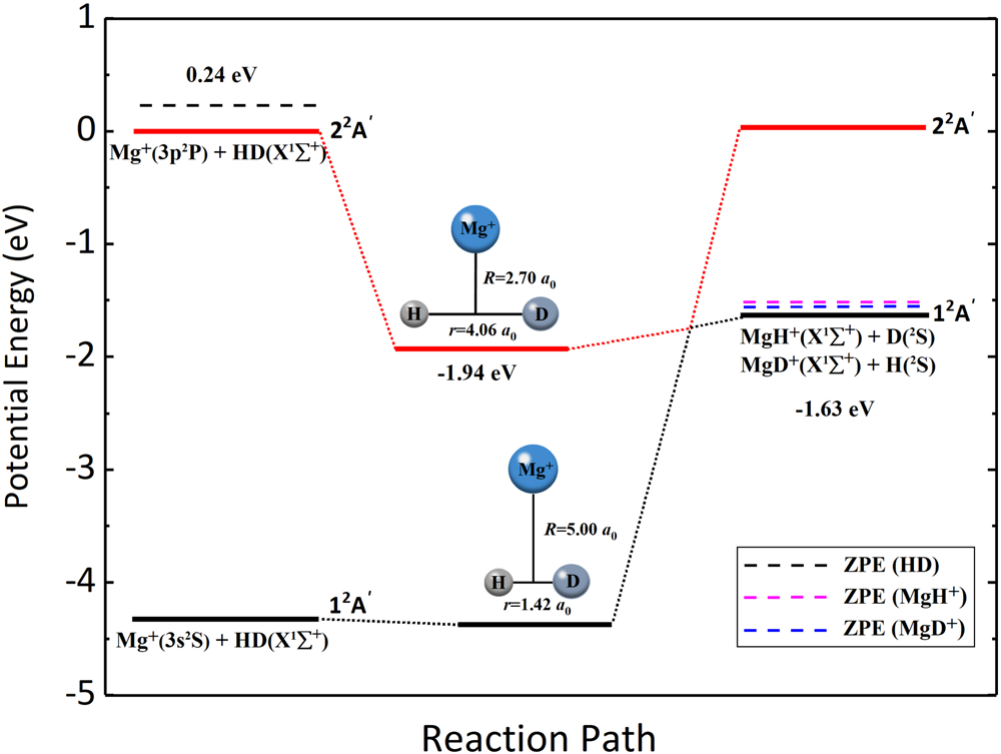
Schematic reaction path for the Mg^+^(3p) + HD reaction on the diabatic representation. The dashed lines represent the ZPEs of HD, MgH^+^ and MgD^+^ molecules.

The dynamics calculations in this paper are based on the YHWCH diabatic PESs of MgH_2_^+^ system^23^. To facilitate the analysis of dynamics results, the schematic reaction path of the Mg^+^(3p) + HD reaction is shown in Fig. 1 according to the YHWCH diabatic PESs. As shown in this figure, the PESs involve with the lowest two adiabatic states 1^2^A′ and 2^2^A′. The reaction of the Mg^+^(3p) + HD starts from the first excited state and then intersects the ground state into the product channel. The title reaction goes through an exothermic process with a large exothermicity of 1.63  eV. The zero-point energy (ZPE) of product molecules MgH^+^ and MgD^+^ is 0.11  eV and 0.08  eV, respectively. When considering the ZPE effect, the exothermicity of the MgH^+^ + D channel and MgD^+^ + H channel is 1.76  eV and 1.79  eV, respectively. In addition, there exists a well along the reaction path, which indicates the presence of insertion collision complex. And the depth corresponding to the reactant channel is 1.94  eV. The combination of complex is mainly dominated by the T-shaped configuration, especially at low collision energies. According to the YHWCH diabatic PESs, this structure at *C*_2v_ symmetry is most stable in the diabatic representation. Besides, it can be seen that the bond length of H-D of the complex in the title reaction is larger than the distance between H and D atoms (1.42  bohr) of the equilibrium structural MgHD^+^ in the ground state. This indicates that there exists an H-D bond-stretch mechanism, which is consistent with previous photofragmentation studies^24,25^. In a word, a clear physical picture about the diabatic process of the Mg^+^(3p) + HD reaction could be concluded as: The excited Mg^+^ ion inserts into the H-D bond to form the MgHD^+^ complex with a stretched H-D bond on the first excited state, and then the complex dissociates into the MgH^+^ or MgD^+^ product through a diabatic transition to the ground state.

**Figure 2:**
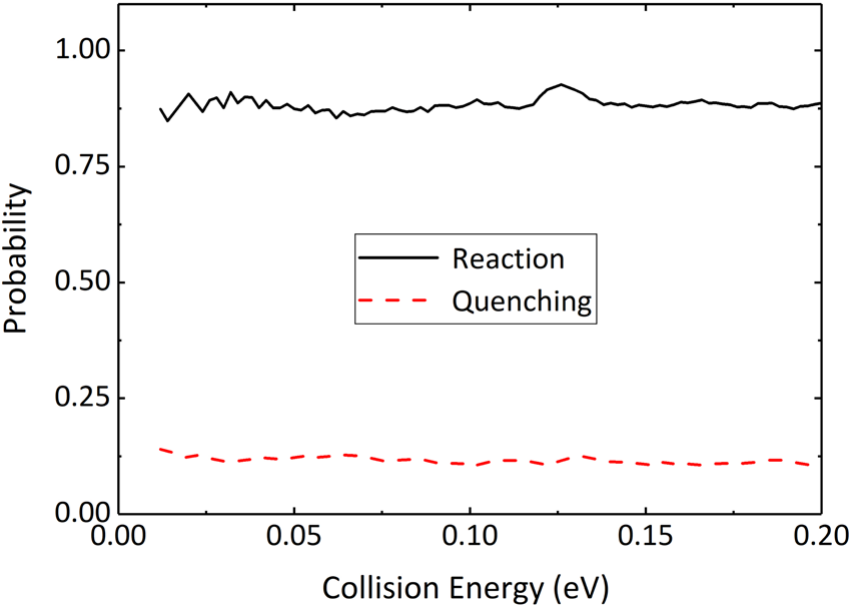
Total reaction and quenching probabilities as a function of collision energy for the Mg^+^(3p) + HD reaction at *J* = 0 partial wave.

Working on this basis, the analyses of the dynamics results involved in the Mg^+^(3p) + HD reaction begin with Fig. 2. As mentioned earlier, because of the region of conical intersection in the PESs, the Mg^+^(3p) + HD → Mg^+^(3s) + HD quenching process is allowed to occur in the entrance channel of the reaction. Figure 2 shows the total probabilities of the reaction process and the quenching process as a function of collision energy for *J* = 0 partial wave. It can be readily seen that, compared with the reaction process, the quenching probabilities are quite small, suggesting that the high efficiency of the reactive process. Similar results in the Mg^+^(3p) + H_2_ reaction have been reported in previous theoretical study^25^. They showed that the reasons for the observed quenching process is not obvious may be because the product molecules MgH^+^ have already been formed in the excited state before the diabatic transition to the ground state.

**Figure 3:**
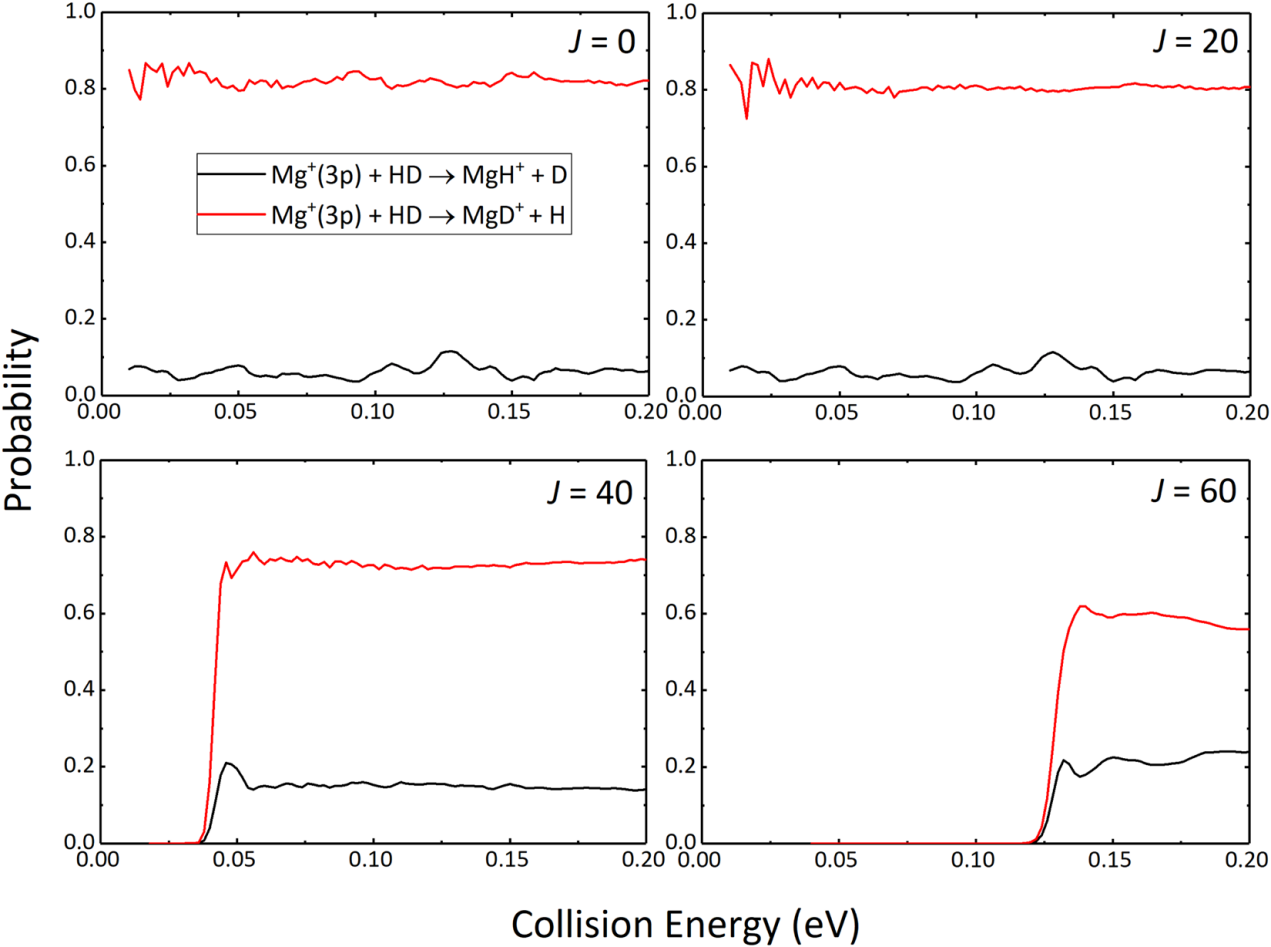
Reaction probabilities of the MgH^+^ + D and MgD^+^ + H product channels as a function of collision energy for selected total angular momentum *J* values (0, 20, 40 and 60).

The reaction probabilities of the Mg^+^(3p) + HD → MgH^+^ + D and Mg^+^(3p) + HD → MgD^+^ + H reaction channels for selected total angular momentum *J* values (0, 20, 40 and 60) are depicted in Fig. 3. As the figure shows, the reaction probabilities of the two reaction channels have some similar features. For *J* = 0, both of the reaction channels do not exist thresholds, which is a typical feature of exothermic reaction without barrier. There exist some resonance structures on the reaction probability curves of the two reaction channels, particularly at low collision energies, which can be attributed to the formation of the intermediate complexes in the potential well on the reaction path. The oscillatory amplitude is relatively weak due to the shallow well and the large exothermicity of the title reaction. The curves tend to be smooth as *J* value increases, indicating that the influence of potential well is weakened due to the emergence of centrifugal energy barrier. Besides, more notably, the reaction probabilities of product MgD^+^ are much greater than that of MgH^+^ at all selected collision energies. This suggests that the MgD^+^ + H channel plays a leading role in the Mg^+^(3p) + HD reaction, which is consistent with previous experimental studies^13^. A detailed discussion regarding the MgD^+^/MgH^+^ branching ratio and the reason of predomination of the MgD^+^ + H channel will be performed below.

**Figure 4:**
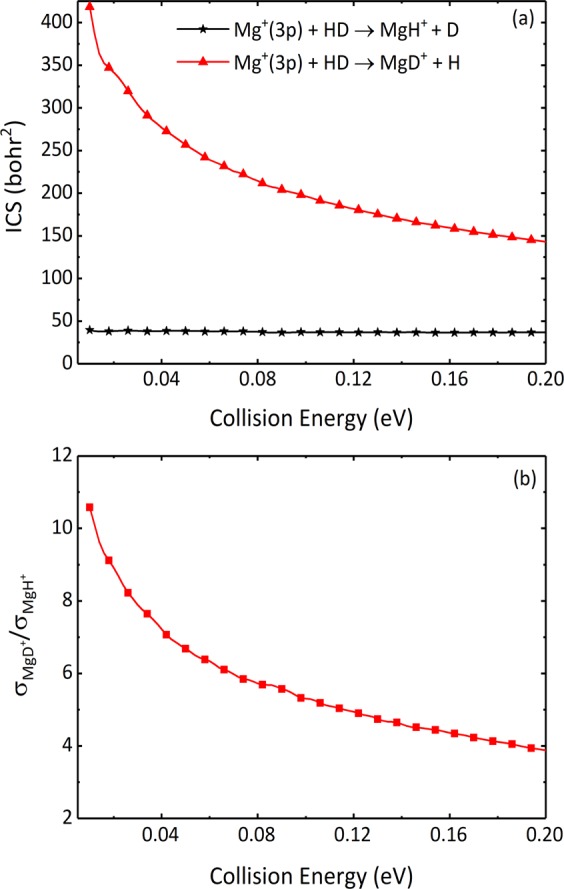
(**a**) Total ICSs of the Mg^+^(3p) + HD → MgH^+^/MgD^+^ + D/H reaction in the collision energy range of 0.01 to 0.20  eV. (**b**) The MgD^+^/MgH^+^ branching ratio as a function of collision energy.

The collision energy dependence of total integral cross sections (ICSs) of the two reaction channels are shown in Fig. 4(a). The total ICS values of the MgD^+^ + H channel are surprisingly higher than that of the MgH^+^ + D channel, notably at low collision energy values. Regarding the MgD^+^ + H channel, the total ICS values decrease significantly as the collision energy increases, which is consistent with the barrierless insertion reaction systems. However, the total ICS of the MgH^+^ + D channel decreases slightly with collision energy, about 39.5 bohr^2^ to 36.9 bohr^2^, owing to its low reactivity. To obtain more sufficient information regarding isotope effects, the MgD^+^/MgH^+^ branching ratio is shown in Fig. 4(b). As seen from the figure, the branching ratio curve is relatively smooth and decreases monotonously as the collision energy increases. The values of MgD^+^/MgH^+^ branching ratio are larger than 3.88 at selected collision energy range, especially it can reach up to 10.58 when the collision energy is 0.01  eV. The high value of the MgD^+^/MgH^+^ branching ratio shows a strong preference of the production of MgD^+^. Besides, the MgD^+^/MgH^+^ branching ratio measured by Staanum and coworkers is greater than 5^13^. In their experiments, the temperature of the HD gas was room temperature, while the Mg^+^ ions were cooled to a temperature much lower than 100  mK. When the collision energy is 0.114  eV, the MgD^+^/MgH^+^ branching ratio calculated in this work is 5.03, which is consistent with the experimental value.

The preference of the MgD^+^ + H channel over the MgH^+^ + D channel is primarily because of the insertion reaction mechanism in the Mg^+^(3p) + HD reaction. The dominance of the XD channel can also be found in other X + HD type insertion reactions, such as the O(^1^D) + HD, C(^1^D) + HD and S(^1^D) + HD. The corresponding branching ratios of XD / XH are roughly 1.35 ± 0.20^26^, 1.6 ± 0.1^27^, and 2.1^28^ at room temperature, respectively. Other similar results are given in refs. ^29–33^. These reactions are known to proceed via a deep potential well and the product molecules are further formed by the long-lived intermediate complex HXD, where X is O(^1^D), C(^1^D) or S(^1^D). Therefore, the lighter H atom is more probably to be ejected from the complex HXD. As discussed above, the Mg^+^ ion inserts into H-D bond and forms the MgHD^+^ complex with a stretched H-D bond, which is beneficial to the formation of MgD^+^. However, compared to the above complex-forming reactions, the preference on the MgD^+^ + H channel of the Mg^+^(3p) + HD reaction is more obvious, it could be because the life of complex is far shorter than the other deep well reactions. Besides, another reason about the preference of the MgD^+^ + H channel may stem from the difference in ZPE of the MgD^+^ and MgH^+^ molecules. The ZPE of MgD^+^ is 0.03  eV smaller than MgH^+^, which results in a larger exothermicity in the MgD^+^ + H channel. Therefore, the reaction prefers to proceed toward the MgD^+^ + H channel because it is easier to react, especially in a very low collision energy region. However, the influence of ZPE effect gradually decreases as the collision energy increases. This causes the advantage of the MgD^+^ + H channel is weakened, and the competitiveness of the MgH^+^ + D channel is enhanced. The above analysis is also applicable to explain the features of ICSs of two channels.

**Figure 5:**
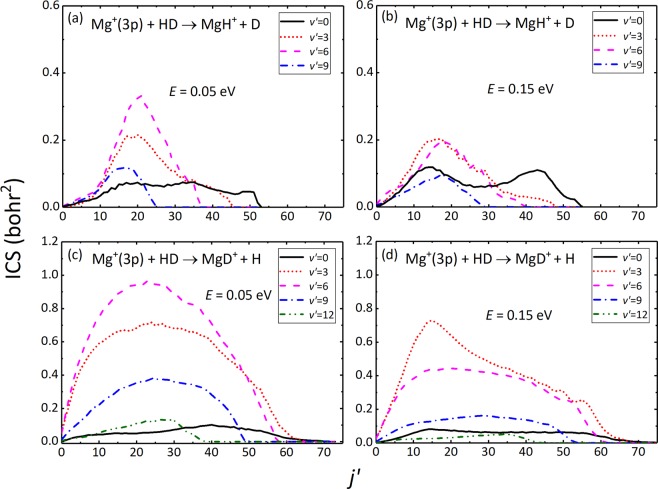
Rovibrational state-resolved ICSs for the Mg^+^(3p) + HD → MgH^+^ + D [(**a**,**b**)] and Mg^+^(3p) + HD → MgD^+^ + H [(**c**,**d**)] reactions at collision energies of 0.05  eV and 0.15  eV.

To obtain more rich dynamical information especially at quantum state-resolved level of the products, the rovibrational state-resolved ICSs of the two products are calculated by TDWP method. For the sake of clarity, only several selected vibrational states are depicted in Fig. 5. As seen from the figure, the products of the two channels both can be excited to extremely high and inverted rovibrational states because of the large exothermicity of the title reaction, and product MgD^+^ is more pronounced. More specifically, the *v*′ = 0 – 9 vibrational states of MgH^+^ are populated, whereas the higher *v*′ = 10, 11 and 12 channels of MgD^+^ are also opened at collision energies of 0.05  eV and 0.15  eV. Compared with the MgH^+^ + D channel, the rotational state distributions of MgD^+^ product are relatively hot and widely distributed. These differences between two products are due to the fact that the vibrational frequency and rotational constant of MgD^+^ are smaller than MgH^+^. The rotational distributions of MgH^+^ and MgD^+^ products become narrow as the increases of products vibrational number *v*′. Additionally, we can also note that there are obvious bimodal rotational state distributions of MgH^+^ at collision energy of 0.15  eV. The bimodal distributions can be attributed to the two different modes of decomposition of MgHD^+^ insertion collision complexes. Similar features were also found for the reactions of the Mg(^1^P_1_) + H_2_/HD/D_2_ previously^34^.

**Figure 6:**
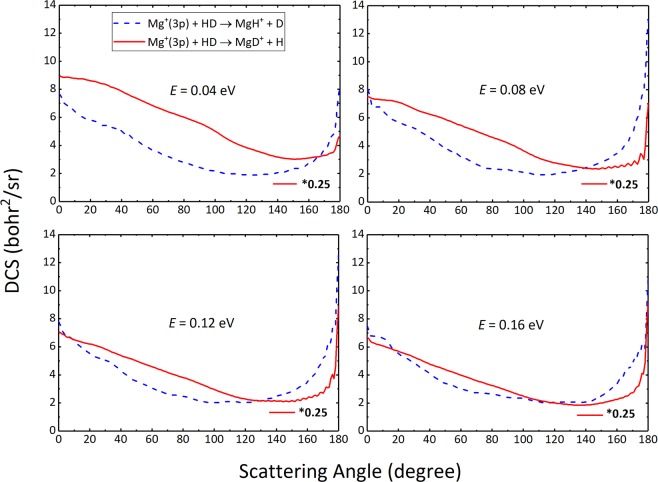
Total DCSs of the Mg^+^(3p) + HD → MgH^+^/MgD^+^ + D/H reaction at four different collision energies. For comparison purposes, the DCSs of the MgD^+^ + H channel are reduced by 4 times.

The differential cross section (DCS) could better reveal the mechanism of chemical reaction. In Fig. 6, the total DCSs of the two reaction channels at four selected collision energies (0.04  eV, 0.08  eV, 0.12  eV and 0.16  eV) are depicted. For ease of comparison studies, the DCS results of the MgD^+^ + H channel are reduced by 4 times. As shown in Fig. 6, both of the two channels exist forward and backward scatterings, which implies that the title reaction is governed by an indirect reactive mechanism. In striking contrast to the MgH^+^ + D channel, the MgD^+^ + H channel has broader forward distributions and the trend of forward scattering gets weaker with the increase of collision energy. This is because the title reaction is dominated by the vertical insertion collision, especially at low collision energies. The consequence of this collision makes the MgD^+^ + H channel have more advantages in the title reaction and usually leads to forward scatterings. However, as the collision energy increases, more angles of insertion collision appear, leading to the weakening of the forward peaks of the MgD^+^ + H channel. In general, unlike the complex-forming insertion reaction with deep well on the reaction path, the DCS distribution of the title reaction does not show strictly forward-backward symmetric trend. The complexes in the reaction have a short lifetime as the consequence of shallow well and large exothermicity. This results in the anisotropy angular distributions in space of the products.

**Figure 7:**
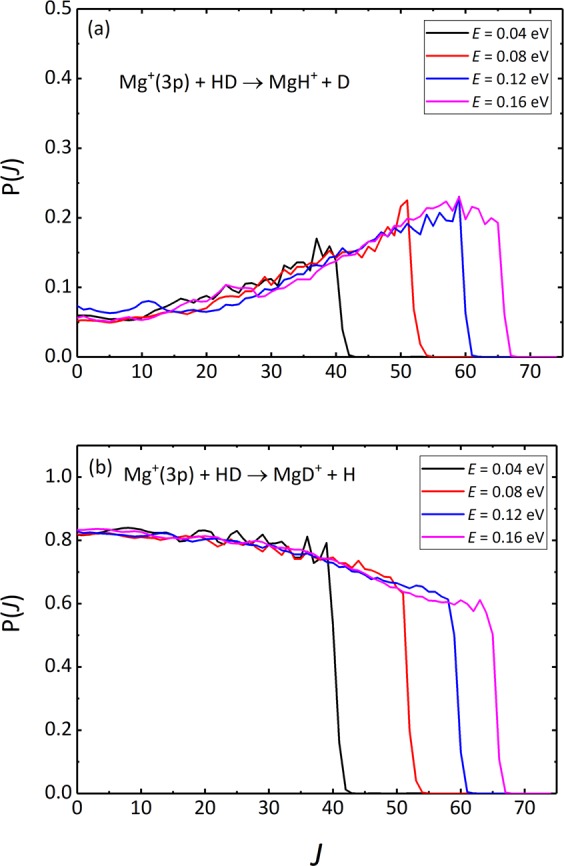
Opacity functions for the Mg^+^(3p) + HD → MgH^+^ + D (**a**) and Mg^+^(3p) + HD → MgD^+^ + H (**b**) reactions at several selected collision energies.

As discussed in the previous studies, there is a direct relationship between the impact parameter *b* and the total angular momentum quantum number *J*^35–37^. Hence, the opacity function P(*J*) and partial DCS can further help us discern the reaction mechanism intuitively. Figure 7 shows the opacity functions of the two channels at several collision energies. It can be seen that the probability functions of the two channels maintain their respective trends until certain *J* values are reached and then fall rapidly at every collision energy. This is because the centrifugal barriers prevent the formation of collision complexes, reducing the probabilities to zero. We also found that the P(*J*) of the MgH^+^ + D channel increases with the increase of *J*, while the MgD^+^ + H channel decreases. This suggests that the MgH^+^ + D channel is dominated by high-*J* partial waves associated with large impact parameters, while the MgD^+^ + H channel is dominated by relatively low-*J* partial waves, which are corresponding to small impact parameters.

**Figure 8:**
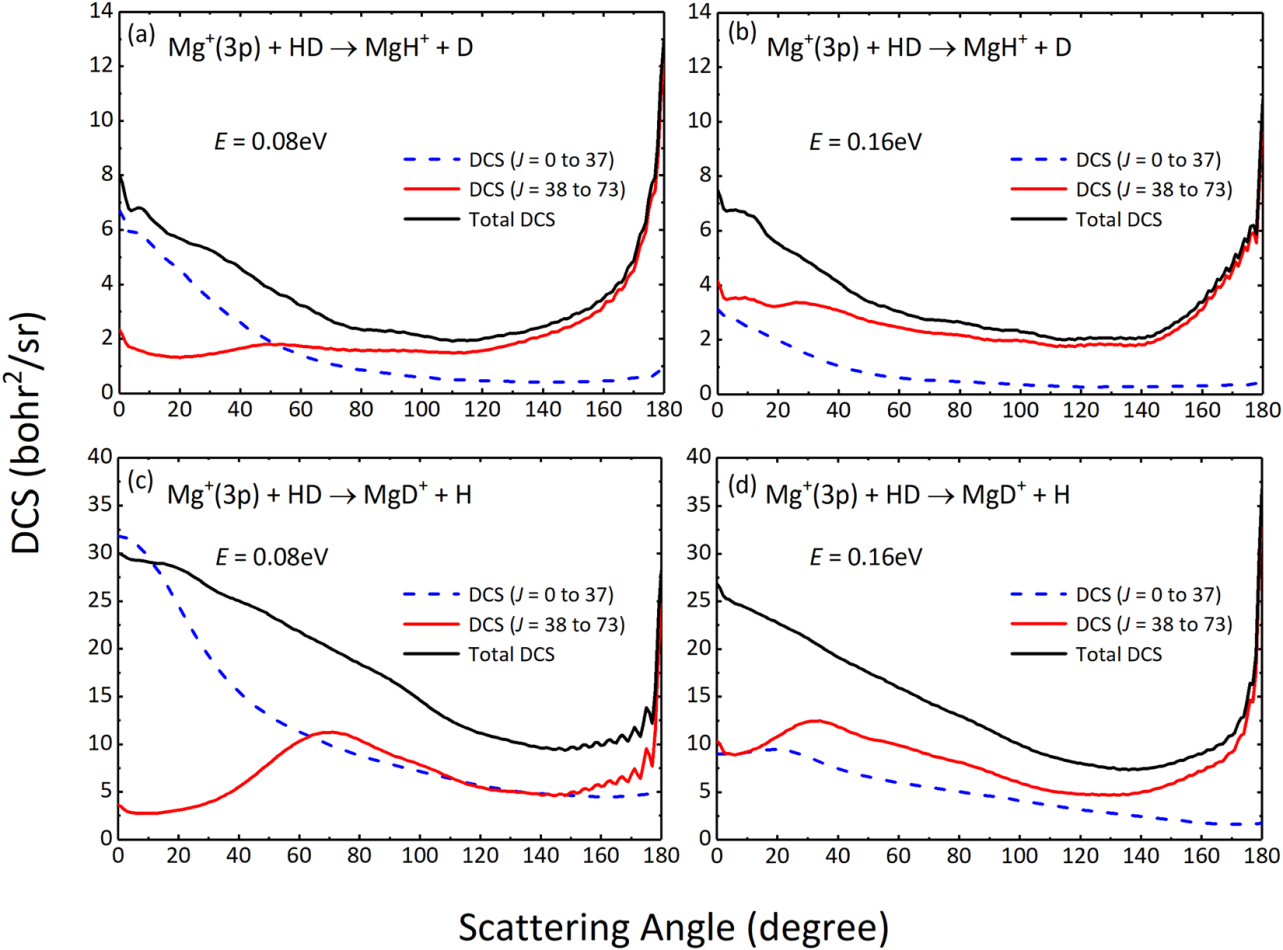
Partial DCSs for the Mg^+^(3p) + HD → MgH^+^ + D [(**a**,**b**)] and Mg^+^(3p) + HD → MgD^+^ + H [(**c,d**)] reactions at collision energies of 0.08  eV and 0.16  eV.

Taking the collision energies of 0.08 and 0.16 as examples, the convergent DCSs correspond to the maximum partial waves are 73. Therefore, half of the maximum *J* value was selected as the dividing value to distinguish the low-*J* (*J* ≤ 37) and high-*J* (*J* ≥38), so as to illustrate the different contributions of partial waves to DCS. The partial DCSs of the two reaction channels are shown in Fig. 8. The results show that the low-*J* partial waves are responsible for the forward scattering part, while the backward scatterings of total DCSs are dominated by high-*J* partial waves. This forward or backward bias means that the reaction is dominated by the formation of short-lived complex. The above results confirm that at low collision energies, the collision way of Mg^+^ nearly direct hitting the center of mass of HD, *i.e*. a smaller impact parameter collision, is more conducive to the formation of MgD^+^, and tends to produce forward scattering. However, as the collision energy increases, the larger impact parameter collisions appear and prevail in the title reaction, resulting in the efficiency of producing MgD^+^ decreases while the MgH^+^ increases.

**Figure 9:**
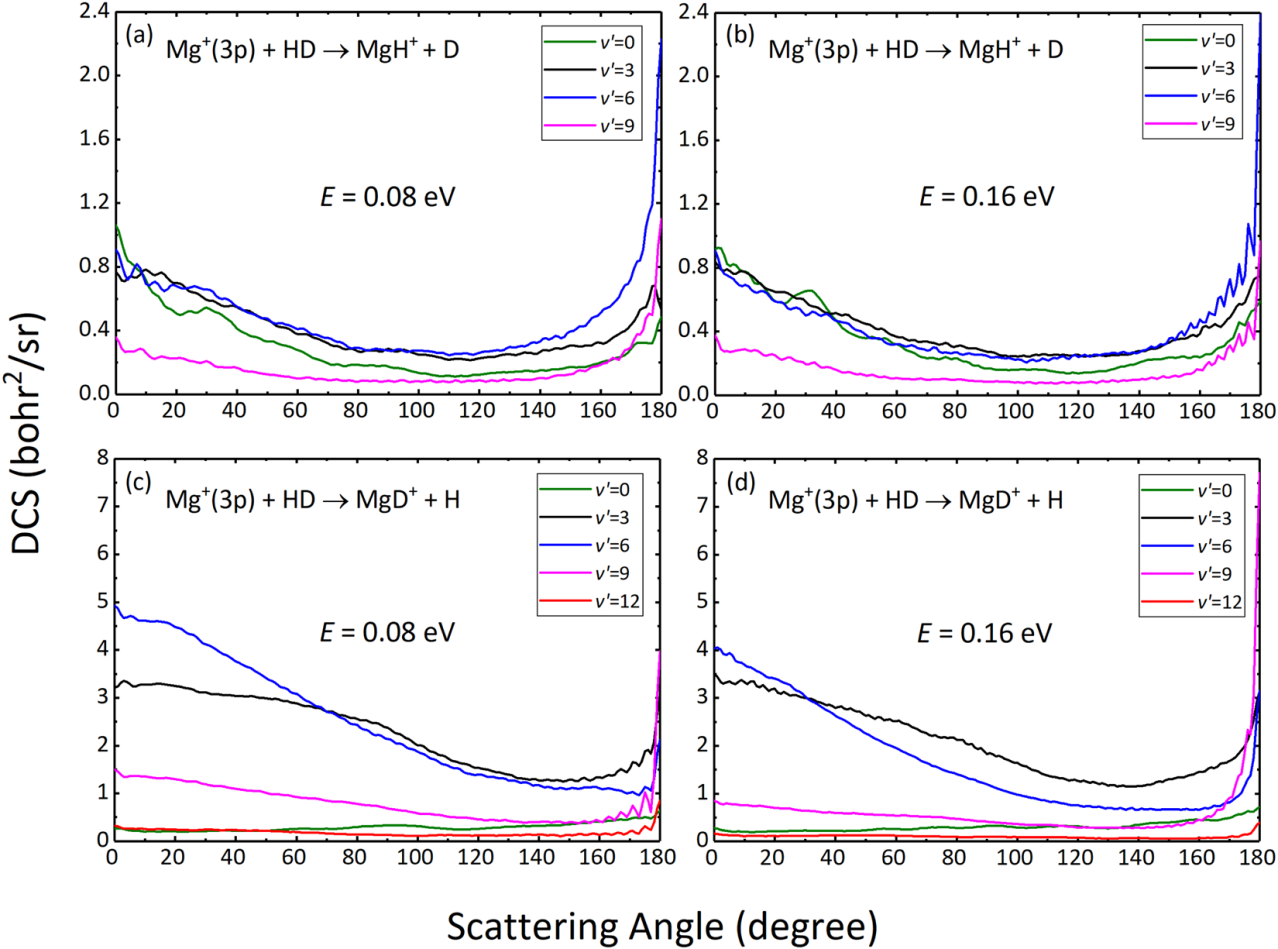
Vibrational state-resolved DCSs of the Mg^+^(3p) + HD → MgH^+^ + D [(**a**,**b**)] and Mg^+^(3p) + HD → MgD^+^ + H [(**c**,**d**)] reactions at collision energies of 0.08  eV and 0.16  eV.

The vibrational state-resolved DCSs of two reaction channels are presented in Fig. 9 at two collision energies of 0.08  eV and 0.16  eV. As seen from the Figure, the relatively high vibrational states of both products correspond to large DCS values, showing obvious population inversion distributions in the vibrational states of the products. The products MgH^+^ in vibrational states with high DCS values tend to backward scatterings dominated by high-*J* partial waves, whereas the products MgD^+^ tend to forward scatterings dominated by low-*J* partial waves. This is in perfect accord with the previous observation of the P(*J*) and partial DCSs. Additionally, we can also note that for backward scatterings, the most efficient energy transfer occurs to the *v*′ = 6 vibrational level of the MgH^+^ products, while extremely high backward peak at 180° of products MgD^+^ appears in *v*′ = 9 state, which is higher than the vibrational state of MgH^+^. The interpretation of this phenomenon can still be based on the consideration of the vibrational frequency of the product, as we discussed in Fig. 5.

## Discussion

In this work, the diabatic dynamics calculations of the Mg^+^(3p) + HD → MgH^+^/MgD^+^ + D/H reaction are performed on the diabatic YHWCH PESs by using the TDWP method. The study takes the significant isotope effect on the title reaction as the starting point, aiming to find and explain the difference between the two reaction channels. The reaction probabilities, ICSs and DCSs of the two channels are calculated and compared. Special attention has been paid to the calculations of the quenching probabilities, but the results show that the quenching process is not obvious compared to the reaction process. The results of reaction probabilities and total ICSs show a dramatic preference to the formation of MgD^+^ over MgH^+^. The calculated MgD^+^/MgH^+^ branching ratio witnesses a monotonic decrease from 10.58 to 3.88 as the collision energy changes from 0.01 to 0.20  eV. When the collision energy is 0.114  eV, the MgD^+^/MgH^+^ branching ratio calculated in this work is close to the experimental value of 5. The main reason of the preference of the MgD^+^ + H channel arises from the insertion reaction mechanism in the title reaction. The Mg^+^ inserts into H-D bond and forms the MgHD^+^ complex with a stretched H-D bond, and eventually favors the formation of MgD^+^. Moreover, the difference in the exothermicity of the two channels caused by the zero-point energies of the product molecules may also be an important factor. The products of MgD^+^ can be excited to higher rovibrational states because of the smaller vibrational frequency and rotational constant of MgD^+^. The opacity function P(*J*) suggests that the most significant disparity between the two channels is mainly due to the contribution of low-*b* collisions at low collision energies. The total and partial DCS results indicate that both forward and backward scatterings exist, which are contributed by low-*J* partial waves and high-*J* partial waves, respectively. Furthermore, several selected vibrational state-resolved DCSs of two reaction channels at collision energies of 0.08  eV and 0.16  eV are also discussed.

## Method

### TDWP

The TDWP method was widely applied in the field of state-to-state reaction dynamics^38–42^. The Mg^+^(3p) + HD → MgH^+^/MgD^+^ + D/H reaction involves two electronic states, and the non-adiabatic couplings can be treated efficiently by using the TDWP method. The detailed theory and implementation of the TDWP method have been discussed in many impressive works^43–45^, and here is only a brief outline for clarity. Reactant Jacobi coordinates (*r, R, θ*) in the body fixed representation are applied in TDWP method, *r* is the HD molecule bond length, *R* is the distance from Mg^+^ ion to the center of mass of the HD molecule and *θ* is the angle between *R* and *r*. The Hamiltonian in this coordinate system can be expressed as follow

1 $$\hat{H}=-\,\frac{{\hslash }^{2}}{2{\mu }_{R}}\frac{{\partial }^{2}}{\partial {R}^{2}}-\frac{{\hslash }^{2}}{2{\mu }_{r}}\frac{{\partial }^{2}}{\partial {r}^{2}}+\frac{{(\hat{J}-\hat{j})}^{2}}{2{\mu }_{R}{R}^{2}}+\frac{{\hat{j}}^{2}}{2{\mu }_{r}{r}^{2}}+\hat{V}$$

where ${\mu }_{R}$ and ${\mu }_{{\rm{r}}}$ represent the corresponding reduced masses for *R* and *r*, respectively. $\hat{J}$ represents the total angular momentum operator, and $\hat{j}$ represents rotational angular momentum operator of the reactant molecule HD. $\hat{V}$ is the potential energy of the system.

The second-order split operator method is employed in the propagation of wave packet. The state-to-state *S-*matrixes of the two product channels are extracted using reaction coordinate-based method. The state-to-state reaction probability can be obtained by the following formula

2 $${P}_{vj\leftarrow {v}_{0}{j}_{0}}^{J}=\frac{1}{2{j}_{0}+1}{\sum _{K,{K}_{0}}|{S}_{vjK\leftarrow {v}_{0}{j}_{0}{K}_{0}}^{J\epsilon }|}^{2}$$

the state-to-state ICSs and DCSs can be obtained using

3 $${\sigma }_{vj\leftarrow {v}_{0}{j}_{0}}=\frac{\pi }{(2{j}_{0}+1){k}_{{v}_{0}{j}_{0}}^{2}}\sum _{K}\sum _{{K}_{0}}\sum _{J}(2J+1){|{S}_{vjK\leftarrow {v}_{0}{j}_{0}{K}_{0}}^{J\epsilon }|}^{2}$$

and

4 $$\frac{d{\sigma }_{vj\leftarrow {v}_{0}{j}_{0}}(\theta ,E)}{d\Omega }=\frac{1}{(2{j}_{0}+1)}{\sum _{K}\sum _{{K}_{0}}|\frac{1}{2i{k}_{{v}_{0}{j}_{0}}}\sum _{J}(2J+1){d}_{K{K}_{0}}^{J}(\theta ){S}_{vjK\leftarrow {v}_{0}{j}_{0}{K}_{0}}^{J\epsilon }|}^{2}$$

where *θ* is the scattering angle.

For the Mg^+^(3p) + HD → MgH^+^/MgD^+^ + D/H reaction, the initial rotational-vibrational state of HD molecule is set as *v*_0_ = 0, *j*_0_ = 0 and all the Coriolis coupling effects are taken into account in the TDWP calculations. The calculated maximum partial wave is *J* = 73 and the upper limit of collision energy corresponding to the convergent ICSs and DCSs is 0.20  eV. The primary parameters used in the TDWP method are listed in Table 1.

**Table 1:** Numerical parameters used in the TDWP calculations.

Mg^+^(3p) + HD → MgH^+^/MgD^+^ + D/H	
Grid/basis range and size	*R* (bohr) $\in$ [0.01, 22.0], ${N}_{R}$ = 329 *r* (bohr) $\in$ [0.01, 16.0], ${N}_{r}$ = 159 ${N}_{j}$ = 99
Initial wave packet $\exp [-\frac{{(R-{R}_{c})}^{2}}{2{\varDelta }_{R}^{2}}]\cos ({k}_{0}R)$	${R}_{0}$ = 16.5 bohr ${\Delta }_{R}$ = 0.30 bohr ${k}_{0}=\sqrt{2{E}_{0}{\mu }_{R}}$ with ${E}_{0}$ = 0.15 eV
Total propagation time	50000 a.u.
Highest *J* value	73

## References

[1] Wang T (2013). Dynamical Resonances Accessible Only by Reagent Vibrational Excitation in the F + HD → HF + D Reaction. Science.

[2] Zhang J, Gao SB, Wu H, Meng QT (2015). State-to-State Quantum Dynamics of Reactions O(^3^P) + HD (*v* = 0 - 1, *j* = 0) → OH + D and OD + H: Reaction Mechanism and Vibrational Excitation. J. Phys. Chem. A.

[3] Yuan JC, Cheng DH, Chen MD (2014). Time-dependent wave packet and quasiclassical trajectory studies of the Au + HD reaction: competition between the reactive channels. Rsc Adv..

[4] Panda AN (2008). Quantum dynamics of Br + HD reaction. J. Phys. Chem. A.

[5] Tiwari AK, Panda AN, Sathyamurthy N (2006). Isotopic branching in (He, HD^+^) collisions. J. Phys. Chem. A.

[6] De Fazio D, Aquilanti V, Cavalli S, Aguilar A, Lucas JM (2008). Exact state-to-state quantum dynamics of the F + HD → HF(*v’* = 2) + D reaction on model potential energy surfaces. J. Chem. Phys..

[7] Chen MD, Tang BY, Han KL, Lou NQ (2001). Quasi-classical trajectory study of the DCl/HCl product branching ratios for the Cl + HD reaction on BW2 potential energy surface. Chem. Phys. Lett..

[8] Yang TG (2015). Extremely short-lived reaction resonances in Cl + HD (*v* = 1) → DCl + H due to chemical bond softening. Science.

[9] Yang BH, Yin HM, Han KL, Zhang JZH (2000). Time-dependent quantum dynamics study of the Cl + HD reaction. J. Phys. Chem. A.

[10] Gao SB, Wei W, Zheng B, Song YZ, Meng QT (2014). Dynamical Properties of S(^3^P) + HD Reaction on 1^3^A” State and Their Quantum Wavepacket Calculation. Int. J. Quantum Chem..

[11] Dalleska NF, Crellin KC, Armentrout PB (1993). Reactions of Alkaline-Earth Ions with H_2_, D_2_, and HD. J. Phys. Chem..

[12] Hansen AK, Sorensen MA, Staanum PF, Drewsen M (2012). Single-Ion Recycling Reactions. Angew. Chem. Int. Ed..

[13] Staanum PF, Hojbjerre K, Wester R, Drewsen M (2008). Probing isotope effects in chemical reactions using single ions. Phys. Rev. Lett..

[14] Baba T, Waki I (2002). Chemical reaction of sympathetically laser-cooled molecular ions. J. Chem. Phys..

[15] Hasegawa T, Shimizu T (2002). Resonant oscillation modes of sympathetically cooled ions in a radio-frequency trap. Phys. Rev. A.

[16] Roth B, Blythe P, Wenz H, Daerr H, Schiller S (2006). Ion-neutral chemical reactions between ultracold localized ions and neutral molecules with single-particle resolution. Phys. Rev. A.

[17] Kimura N (2011). Sympathetic crystallization of CaH^+^ produced by a laser-induced reaction. Phys. Rev. A.

[18] Sawyer BC, Bohnet JG, Britton JW, Bollinger JJ (2015). Reversing hydride-ion formation in quantum-information experiments with Be^+^. Phys. Rev. A.

[19] Molhave K, Drewsen M (2000). Formation of translationally cold MgH^+^ and MgD^+^ molecules in an ion trap. Phys. Rev. A.

[20] Bauschlicher CW (1993). The Ground and Low-Lying Excited-States of MgH_2_^+^. Chem. Phys. Lett..

[21] Dryza V, Bieske EJ, Buchachenko AA, Klos J (2011). Potential energy surface and rovibrational calculations for the Mg^+^-H_2_ and Mg^+^-D_2_ complexes. J. Chem. Phys..

[22] Satta M, Marquez-Mijares M, Yurtsever E, Bovino S, Gianturco FA (2013). Mg^+^(^2^S) and Mg^+^(^2^P) in reaction with H_2_(^1^Σ_g_^+^): A description of the energy surfaces explaining the mechanisms. Int. J. Mass Spectrom..

[23] Yuan JC, He D, Wang SF, Chen MD, Han KL (2018). Diabatic potential energy surfaces of MgH_2_^+^ and dynamic studies for the Mg^+^(3p) + H_2_ → MgH^+^ + H reaction. Phys. Chem. Chem. Phys..

[24] Ding LN, Young MA, Kleiber PD, Stwalley WC, Lyyra AM (1993). Photofragmentation Spectroscopy of MgD_2_^+^. J. Phys. Chem..

[25] Kleiber PD, Chen J (1998). Spectroscopy and chemical dynamics of weakly bound alkaline-earth metal ion-H_2_ and alkaline-earth metal ion-hydrocarbon complexes. Int. Rev. Phys. Chem..

[26] Laurent T (1995). Absolute Rate Constants, Reactive Cross-Sections and Isotopic Branching Ratio for the Reaction of O(^1^D) with HD. Chem. Phys. Lett..

[27] Sato K, Ishida N, Kurakata T, Iwasaki A, Tsunashima S (1998). Reactions of C(^1^D) with H_2_, HD and D_2_: kinetic isotope effect and the CD/CH branching ratio. Chem. Phys..

[28] Lin SL, Guo H (2005). Quantum statistical and wave packet studies of insertion reactions of S(^1^D) with H_2_, HD, and D_2_. J. Chem. Phys..

[29] Hankel M, Balint-Kurti GG, Gray SK (2001). Quantum mechanical calculation of reaction probabilities and branching ratios for the O(^1^D) + HD → OH(OD) + D(H) reaction on the X̃^1^A’ and 1^1^A” adiabatic potential energy surfaces. J. Phys. Chem. A.

[30] Hsu YT, Wang JH, Liu KP (1997). Reaction dynamics of O(^1^D) + H_2_, D_2_, and HD: Direct evidence for the elusive abstraction pathway and the estimation of its branching. J. Chem. Phys..

[31] Sun ZP, Zhao WK, Yang CL (2017). Quantum reaction dynamics of C(^1^D) + HD → CH(CD) + D(H) on the ground state potential energy surface. Int. J. Quantum Chem..

[32] Tsukiyama K, Katz B, Bersohn R (1985). Isotopic Branching Ratio for the Reaction A + HD → AD(H) + H(D). J. Chem. Phys..

[33] Yang HA, Han KL, Schatz GC, Smith SC, Hankel M (2010). Exact and truncated Coriolis coupling calculations for the S(^1^D) + HD reaction employing the ground adiabatic electronic state. Phys. Chem. Chem. Phys..

[34] Breckenridge WH, Wang JH (1987). Dynamics of the Reactions of Mg(3s3p^1^P_1_) with H_2_, HD, and D_2_: Rotational Quantum State Distributions of MgH (MgD) Products. Chem. Phys. Lett..

[35] Jambrina PG, Menendez M, Aoiz FJ (2018). Angular momentum-scattering angle quantum correlation: a generalized deflection function. Chem. Sci..

[36] Xie CJ, Liu XG, Guo H (2018). State-to-state quantum dynamics of the H + LiF → Li + HF reaction on an accurate *ab initio* potential energy surface. Chem. Phys..

[37] Jambrina PG (2010). The dynamics of the H^+^ + D_2_ reaction: a comparison of quantum mechanical wavepacket, quasi-classical and statistical-quasi-classical results. Phys. Chem. Chem. Phys..

[38] Yang ZJ, Yuan JC, Wang SF, Chen MD (2018). Global diabatic potential energy surfaces for the BeH_2_^+^ system and dynamics studies on the Be^+^(^2^P) + H_2_(X^1^Σ_g_^+^) → BeH^+^(X^1^Σ_g_^+^) + H(^2^S) reaction. Rsc Adv..

[39] Wang SF, Yang ZJ, Yuan JC, Chen MD (2018). New diabatic potential energy surfaces of the NaH_2_ system and dynamics studies for the Na(3p) + H_2_ → NaH + H reaction. Sci. Rep..

[40] He D, Yuan JC, Chen MD (2017). Influence of rovibrational excitation on the non-diabatic state-to-state dynamics for the Li(2p) + H_2_ → LiH + H reaction. Sci. Rep..

[41] Wu H, Liang DY, Zhang PY (2015). Time-dependent wave packet state-to-state quantum dynamics study of the abstraction reaction S(^3^P) + H_2_(*v* = 0, *j* = 0) on 1^3^A” electronic state. Chem. Phys..

[42] Wang SF, Yuan JC, Li HX, Chen MD (2017). A neural network potential energy surface for the NaH_2_ system and dynamics studies on the H(^2^S) + NaH(X^1^Σ^+^) → Na(^2^S) + H_2_(X ^1^Σ_g_^+^) reaction. Phys. Chem. Chem. Phys..

[43] Sun ZG, Lee SY, Guo H, Zhang DH (2009). Comparison of second-order split operator and Chebyshev propagator in wave packet based state-to-state reactive scattering calculations. J. Chem. Phys..

[44] Sun ZG, Guo H, Zhang DH (2010). Extraction of state-to-state reactive scattering attributes from wave packet in reactant Jacobi coordinates. J. Chem. Phys..

[45] Guo H (2012). Quantum dynamics of complex-forming bimolecular reactions. Int. Rev. Phys. Chem..

